# Fatal 1918 Pneumonia Case Complicated by Erythrocyte Sickling

**DOI:** 10.3201/eid1612.101376

**Published:** 2010-12

**Authors:** Zong-Mei Sheng, Daniel S. Chertow, David M. Morens, Jeffery K. Taubenberger

**Affiliations:** Author affiliation: National Institutes of Health, Bethesda, Maryland, USA

**Keywords:** Bacteria, respiratory infections, Streptococcus pneumoniae, pneumonia, influenza, 1918 pandemic influenza, sickle cell anemia, case report, history, letter

**To the Editor:** The year 2010 marks the 100th anniversary of Herrick’s original case description of what is now known as sickle cell anemia (*1*). Additional case reports followed in 1911 and 1915; in 1922, Mason described a fourth case and coined the term sickle cell anemia (*2*). In 1949, Pauling et al. published an important study that identified differences in the electrophoretic mobilities of normal and sickled erythrocytes (*3*). The inheritance pattern of sickle cell anemia was determined in 1949; in 1957, Ingram identified the single amino acid change in hemoglobin S (*4*).

Patients with sickle cell anemia are at markedly increased risk for infections with several bacteria, including *Streptococcus pneumoniae*, and emerging viral infections such as pandemic influenza. We report a retrospectively identified case of fatal bacterial pneumonia complicated by abundant erythrocyte sickling in a patient bearing the sickle cell trait. The patient’s illness occurred in July 1918, representing one of the first identified symptomatic cases of sickle cell trait.

The patient, a 21-year-old African American male, was a US Army private admitted to the post hospital in Fort Riley (Camp Funston), Kansas, USA, on July 11, 1918, >1 month before the first recognized cases of the fall wave of the influenza pandemic in the United States. The patient had a 2-day history of fever, headache, chest pain, and a dry, hacking, nonproductive cough. Medical history consisted only of frequent headaches. Admission temperature was 105.4°F. Physical examination found indistinct breath sounds over the entire right lung. Lobar pneumonia was diagnosed in the patient. On July 15, his leukocyte count was 7,600 cells/mm^3^, and physical examination found crepitant rales over the right lung and tubular breathing over the right upper lobe. On July 19, his condition was grave. Probably because most US military camps had experienced epidemics of measles with fatal streptococcal pneumonia during winter 1917–spring 1918, he was given 100 mL anti-streptococcus antiserum intravenously. He died July 20.

Postmortem examination on July 21 indicated marked consolidation of both the right middle and lower lung lobes, which appeared hemorrhagic. Large areas of right upper lobe necrosis were mixed with areas corresponding grossly to lobar pneumonia. The right pleural cavity was obliterated by fibrinous adhesions. Postmortem cultures from the right upper and lower lung lobes were positive for *S. pneumoniae* type II. Pleural cavity and heart blood cultures were negative. Notable findings included an enlarged hemorrhagic and necrotic spleen and numerous small hemorrhages in the medullary regions of the kidneys. Microscopic results were absent from the available postmortem examination record.

Two hematoxylin and eosin–stained lung sections from this patient, examined by using material from the archives of the Armed Forces Institute of Pathology (*5*) as part of a review of possible 1918 influenza virus pneumonia cases, showed acute pneumonia with extensive necrosis (Technical Appendix Figure, panel A). Brown and Hopps tissue Gram stain revealed abundant gram-positive cocci (Technical Appendix Figure, panel B). Histologic examination found abundant sickled erythrocytes in small pulmonary vessels (Technical Appendix Figure, panels C, D). Results of real-time reverse transcription–PCR for influenza virus matrix 1 gene (*5*) were negative, as were immunohistochemical examination results for influenza viral antigen (Technical Appendix Figure, panel F); control immunohistochemical examination results for cytokeratins were positive (Technical Appendix Figure, panel E).

DNA was extracted from 1 of the formalin-fixed, paraffin-embedded lung blocks. Partial sequence of the hemoglobin beta gene was performed with PCR primer sets designed to span portions of the open reading frame (primers available upon request). Sequence of multiple clones across the gene showed the patient to be heterozygous for the Glu6Val hemoglobin S mutation (*6*), with 1 wild type and 1 mutant allele (Technical Appendix Figure, panels G and H), indicative of sickle cell trait. Sequence analyses for the mutations associated with hemoglobin C (Glu6Lys), hemoglobin D (Glu121Gln), and hemoglobin O (Glu121Lys) showed only wild-type sequence (data not shown).

Although the material had been examined for possible influenza infection, the timing of the illness makes influenza an unlikely cofactor because epidemiologic records show no evidence of influenza or excess deaths from respiratory disease at Fort Riley in July 1918 (*7*). We found no evidence of influenza A viral RNA by reverse transcription–PCR or viral antigen by immunohistochemical examination.

Sickle cell trait has been occasionally associated with debilitating illness and death. Pulmonary complications associated with sickle cell trait include venous thromboembolic disease, sickle chest syndrome (*8*), and pulmonary infarction (*9*), which recently prompted US college officials to screen athletes for sickle cell trait (*10*). We speculate that the clinical severity and rapid development of acute bacterial pneumonia in the patient reported here led to profound terminal hypoxemia, which led in turn to erythrocyte sickling. The postmortem gross evidence of necrotic areas in the lung and spleen and hemorrhages in the kidneys is clearly consistent with sickled erythrocytes causing vascular congestion and infarction, thus contributing to the patient’s death.

## Supplementary Material

Technical Appendix Histopathologic changes associated with a 1918 type II pneumococcal pneumonia case and DNA sequence of a portion of the hemoglobin beta gene..
