# Supplementary material for: Fatal 1918 Pneumonia Case Complicated by Erythrocyte Sickling
Source: Emerg Infect Dis. 2010 Dec;16(12):2000–1. doi: 10.3201/eid1612.101376 (PMC3294594; doi:10.3201/eid1612.101376)
Supplement: Technical Appendix — Histopathologic changes associated with a 1918 type II pneumococcal pneumonia case and DNA sequence of a portion of the hemoglobin beta gene.. [file 10-1376-Techapp_1p.pdf]

Article DOI: 10.3201/eid1612.101376

Fatal 1918 Pneumonia Case Complicated by Erythrocyte Sickling

Technical Appendix

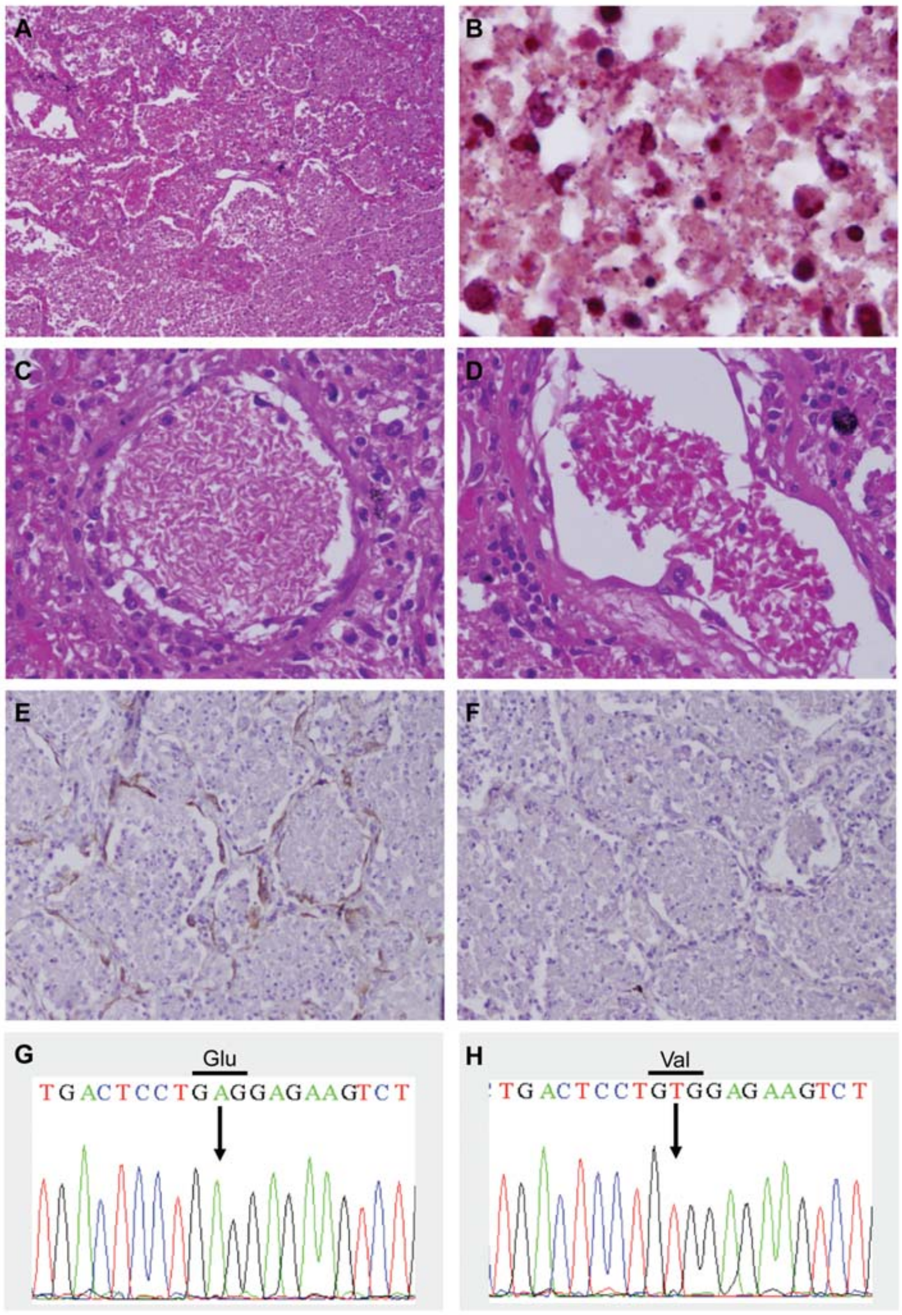

Technical Appendix Figure. Histopathologic changes associated with a 1918 type II pneumococcal pneumonia case and DNA sequence of a portion of the hemoglobin beta gene. A) Hematoxylin and eosin (H&E)–stained lung showing severe acute bacterial pneumonia with tissue necrosis (original magnification ×40). B) Brown and Hopps tissue Gram stained– lung showing abundant gram-positive cocci consistent with pneumococci (original magnification ×1,000). C) Photomicrograph of H&E-stained section showing abundant erythrocyte sickling in a venule (original magnification ×200). D) Photomicrograph of H&E-stained section showing abundant erythrocyte sickling in a venule (original magnification ×400). E) Immunohistochemical stain for cytokeratins in a section of lung. Cytokeratin is red-brown on a hematoxylin-stained background (original magnification ×100). F) Section of lung (original magnification ×100) with immunohistochemical stain for influenza. G) DNA sequence of cloned DNA from a PCR product showing the sequence GAG at position 7 of the hemoglobin beta gene. H) DNA sequence of another PCR product clone showing the hemoglobin S mutation GTG at position 7 of the hemoglobin beta gene.
